# Fractional flow reserve-guided complete revascularization versus culprit-only revascularization in acute ST-segment elevation myocardial infarction and multi-vessel disease patients: a meta-analysis and systematic review

**DOI:** 10.1186/s12872-019-1022-6

**Published:** 2019-03-01

**Authors:** Li-jie Wang, Shuo Han, Xiao-Hong Zhang, Yuan-Zhe Jin

**Affiliations:** Department of Cardiology, the fourth Affiliated Hospital of China Medical University, Shenyang, 110032 Liaoning China

**Keywords:** Fractional flow reserve, Complete revascularization, Culprit-only revascularization, ST-segment elevation myocardial infarction, Multi-vessel disease

## Abstract

**Background:**

Approximately 30–50% patients with acute ST-segment elevation myocardial infarction (STMEI) were found to have non-infarct-related coronary artery (IRA) disease, which was significantly associated with worse prognosis. However, challenges still remain for these patients: which non-infarct-related lesion should be treated and when should the procedure be performed? The present study aims to investigate Fractional flow reserve (FFR)-guided complete revascularization (CR) in comparison to culprit-only revascularization (COR) in patients with ST-segment elevation myocardial infarction (STEMI) and multi-vessel disease (MVD).

**Methods:**

Three appropriate randomized controlled trials (RCTs) were selected from the PubMed/Medline, EMBASE, and the Cochrane library /CENTRAL databases. 1631 patients (688 patients underwent FFR-guided CR and 943 patients underwent COR) following-up 12–44 months was evaluated.

**Results:**

FFR-guided CR significantly reduced major adverse cardiac event (MACE) (OR 0.47, 95% CI: 0.35–0.62, *P* < 0.00001) and ischemia-driven repeat revascularization (OR 0.36, 0.26–0.51, P < 0.00001), as compared to COR. However, there is no difference in all-cause mortality (OR 1.24, 0.65–2.35, *P* = 0.51).

**Conclusions:**

In patients with STEMI and MVD, FFR-guided CR is better than COR in terms of MACE and ischemia-driven repeat revascularization, while there are almost similar in all-cause mortality.

**Trial registration:**

All analyses were based on previous published studies, thus no ethical approval and patient consent are required COMPARE-ACUTE trial number NCT01399736; DANAMI-3–PRIMULTI trial number NCT01960933.

**Electronic supplementary material:**

The online version of this article (10.1186/s12872-019-1022-6) contains supplementary material, which is available to authorized users.

## Background

Approximately 30–50% patients with acute ST-segment elevation myocardial infarction (STMEI) were found to have non-infarct-related coronary artery (IRA) disease, which was significantly associated with worse prognosis [1, 2]. However, challenges still remain for these patients: which non-infarct-related lesion should be treated and when should the procedure be performed? Previously, many STEMI guidelines from AHA/ACC/ESC didn’t recommend to offer complete revascularization for STEMI patients with multi-vessel disease during primary percutaneous coronary intervention (PCI) without hemodynamic instability, which could increase the rate of mortality [3–5]. 2015 ACC/AHA guideline declared IIb recommendation for complete revascularization in selected STEMI patients with multi-vessel disease. Recently, for these patients, the updated 2017 ESC STEMI management guideline recommended complete revascularization that non-infarcted related artery lesion should be treated during either index procedure or index admission, following culprit lesion revascularization. This recommendation was based on the data from PRAMI, DANAMI-3-PRIMULTI, CVLPRIT and COMPARE-ACUTE trials, which favored the reductions in the risk of major adverse cardiovascular event (MACE) and repeat revascularization, not in all-cause or cardiovascular mortality rate. It is important to note that most of the time the evaluation of non-culprit lesion by angiography may not be accurate, because of underestimating or overestimating the lesion. Fractional flow reserve (FFR) can functionally evaluate the pathophysiological significance of the non-culprit lesion by using pressure wire in favor of functional angioplasty [6]. Moreover, the data from Fraction Flow Reserve Versus Angiography for Multi-vessel Evaluation (FAME) study at two years, showed FFR-guided PCI in patients with stable coronary artery disease lowered mortality rate and the rate of re-infarction, compared to angiography-guided PCI [7]. Therefore, we aimed to investigate whether FFR-guided functionally complete revascularization with PCI in patients with STEMI and multi-vessel disease could further improve the prognosis, especially the hard end point.

## Methods

For this meta-analysis, we searched the PubMed/Medline, EMBASE, and the Cochrane library /CENTRAL databases and selected published RCTs which compared FFR-guided CR and COR in STEMI patients with multi-vessel disease up to May 12, 2018. The search terms included “Acute ST-segment elevation Myocardial Infarction”, “STEMI”, “Fractional flow reserve”, “FFR”, “Percutaneous Coronary Intervention”, “PCI”, “complete revascularization”, “culprit-only revascularization”, “Multi-vessel disease”, “culprit lesion” and “non-culprit lesion”. Additionally, presentations and abstracts were also searched from major cardiovascular conferences.

The inclusion criteria were: (1) published Randomized Controlled Trials (RCTs); (2) comparing FFR-guided complete revascularization PCI with culprit only revascularization PCI; (3) a study population of STEMI patients with multi-vessel disease.

The exclusion criteria were: (1) hemodynamic instability, such as cardiogenic shock; (2) previous meta-analysis or overlapping data.

Independently, according to the inclusion and exclusion criteria, three authors (L.J.W, S.H and X.H.Z) assessed RCTs eligibility and bias risk (Additional file 1: Figure S1 and Additional file 2: Figure S2), and extracted data. Their disagreements would be resolved by consensus.

The outcomes involved in the current study were major adverse cardiovascular events (MACE), all-cause mortality, myocardial infarction (MI) and repeated revascularization.

The current meta-analysis followed the PRISMA (Preferred Reporting Items for Systematic Reviews and Meta-Analyses) study guideline [8]. Heterogeneity among the subgroups was estimated by the Cochrane Q-statistic test and I^2^-statistic test [9], whereby a *P*-value > 0.05 implied no statistically different result and a I^2^ value <50% suggested a fixed effect model by using funnel plots assessed publication bias. Meta-analysis were carried out with Review Manager (RevMan) version 5.3(Copenhagen: The Nordic Cochrane Centre, The Cochrane Collaboration, 2014) software, obtaining Odds Ratios (OR) and 95% Confidence Intervals (CIs). a *P* value <0.05 indicated a statistically significant result.

## Results

### Search result

**Fig. 1:**
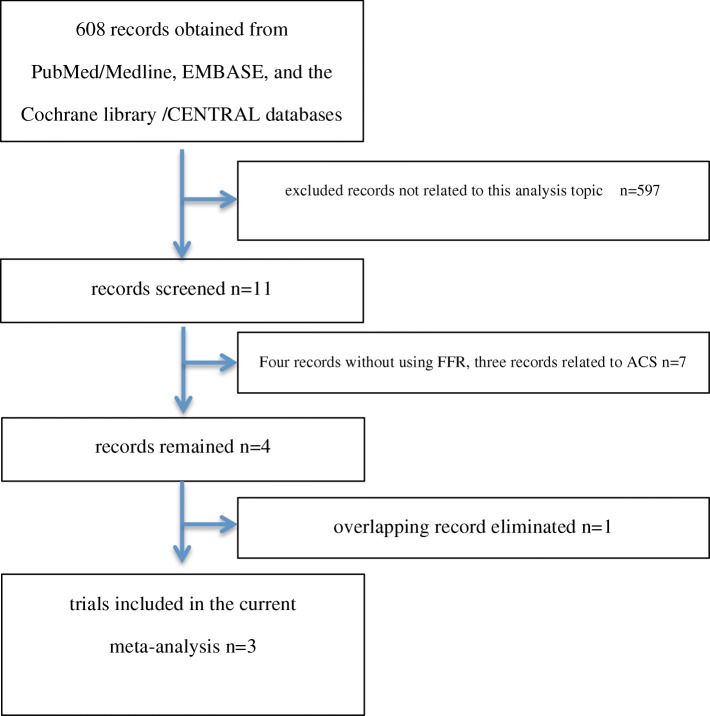
Flow chart representing the study selection

Initially, our search retrieved 608 records and 11 records were found. After reviewing, four records without using FFR [10–13] and three records related to ACS [14–16] were eliminated. According to the primary selection criteria, four records were obtained. Due to two of them were from the same study [17, 18], we excluded 1 trial and obtained three RCTs totally [17, 19, 20]. The study selection flow diagram was shown in Fig. 1.

### Study characteristics and patient features

The current meta-analysis totally included 1631 patients (688 patients underwent FFR-guided CR and 943 patients underwent COR), with 12–44 months follow-up. General features of each study were represented in Table 1, including the number of patients, the cut-off value of FFR, the intervention time of non-culprit lesion, stent type, study time, follow-up term and medical treatment.

**Table 1:** Characteristics of the included studies

No. auther/study	COR	CR	timing of non-IRA intervention	Indication of non-IRA intervention	timing of study	number of centers	primary endpoint	secondary endpoint	stent type	ollow-up	Treatment
1. Ghani et al. [17]	41	80	During the in-hospital phase after primary PCI or in an outpatient setting but no later than 3 weeks after STEMI	FFR < 0.75 Or Diameter stenosis> 90%	From June 2004 to February 2007	Single center	Ejection fraction at 6 months	MACE including death, non-fatal re-infarction, and additional revascularization	BMS 68% DES 20% PTCA12%	3 years	During procedure: glycoprotein IIb/IIIa inhibitors(45%). At discharge: not mentioned
2.DANAMI-3- PRIMULTI [19]	313	314	Two days after the initial PCI procedure and before discharge	FFR ≤ 0.80 Or Diameter stenosis> 90%	From March 2011 to February 2014	2 centers	A composite of all-cause mortality, re-infarction, and ischemia-driven revascularization in the non-IRA	Components of the primary endpoint, cardiac death, and PCI in the non-IRA	BMS 1.5% DES 94% PTCA5%	27 months (12–44 months)	During procedure: glycoprotein IIb/IIIa inhibitors(20%), bivalirudin(75%). At discharge: clopidogrel 12–14% Prasugrel 62–65% Ticagrelor 21–23%
3.COMPAREACUTE [20]	590	590,295	During index PCI procedure (83.4%) Delayed during index hospitalization (16.6%)	FFR ≤ 0.80	From July 2011 to October 2015	24 centers	the composite of all-cause mortality, nonfatal myocardial infarction, any revascularizatio n, and cerebrovascular	Components of the primary endpoints at 24,36 month s	BMS 0.6% DES 98.8% PTCA0.6%	36 months	During procedure: not mentioned At discharge: accordance with contemporary guidelines.

The baseline features of the patients involved in the current meta-analysis were showed in Table 2. The mean age (from 61 to 64 years) of the patients in each study was almost the same. The percentage of male patients in each study was similar, approximately 80%. DANAMI-3- PRIMULTI [19] and COMPARE-ACUTE [20] trial had a slightly higher rate of hypertension, Diabetes mellitus, dyslipidemia and Three-vessel disease than Ghani trial [17]. The rate of smoking and Killip class II–IV in each study was nearly the same.

**Table 2:** Baseline features of patients in the trials involved

Features	Ghani et al. [17]	DANAMI-3- PRIMULTI [19]	COMPARE-ACUTE [20]
	CR/COR	CR/COR	CR/COR
Mean age (year)	62/61	64/63	62/61
Males(%)	80.0/80.5	80/81	79.0/76.3
Hypertension	26.3/42.5	41/47	46.1/47.8
Dyslipidemia(%)	15.0/30.0	–	32.2/29.8
Smoking(%)	44.2/47.5	51/48	40.8/48.7
Diabetes mellitus(%)	6.3/5.0	9/13	14.6/15.9
Three-vessel disease(%)	25.0/19.5	31/32	30.8/32.9
Killip class II–IV	6.3/2.4	7/6	5.1/5.1

### Analysis results

**Fig. 2:**
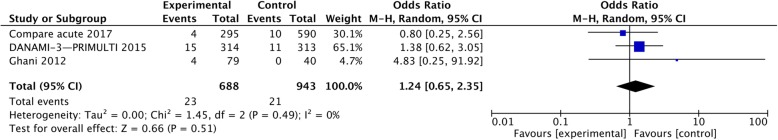
Forest plot of all-cause mortality

The incidence of all-cause mortality did not reveal statistically significant difference between FFR-guided CR group and COR group (3.3% vs. 2.2%; OR: 1.24, 95% CI: 0.65–2.35; *P* = 0.51) (Fig. 2). Furthermore, no significant heterogeneity was showed (I^2^ = 0%) among these trials (Fig. 2).

**Fig. 3:**
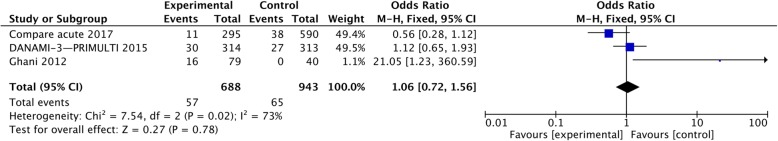
Forest plot of all-cause mortality and MI

The incidence of all-cause mortality and MI was 8.5% in FFR-guided CR group versus 6.9% in COR group, which did not show any statistically significant difference between the two groups (OR: 1.06, 95% CI: 0.72–1.56; *P* = 0.78) (Fig. 3). Among trials moderate significant heterogeneity (I^2^ = 73%) was found (Fig. 3).

**Fig. 4:**
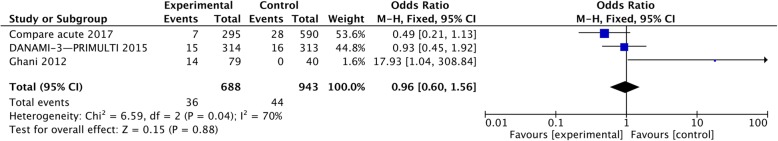
Forest plot of non-fatal MI

The incidence of non-fatal MI was also not significantly different between FFR-guided CR group and COR group (5.2% vs. 4.6%; OR: 0.96, 95% CI: 0.60–1.56; *P* = 0.88) (Fig. 4). The heterogeneity (I^2^ = 70%) among trials was also moderate significant (Fig. 4).

**Fig. 5:**
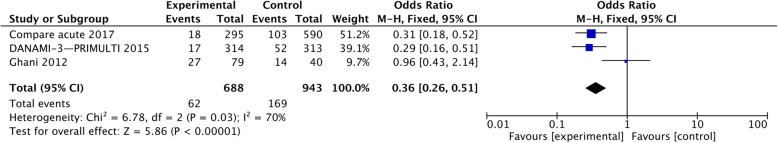
Forest plot of Repeat revasularization

**Fig. 6:**
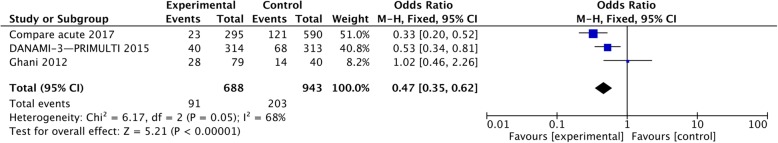
Forest plot of MACE

It is a remarkable fact that the incidence of repeat revascularization or major adverse cardiovascular event (MACE, comprising all-cause mortality, myocardial infarction and repeated revascularization here) shows a statistically significant difference between FFR guided CR group and COR group, respectively (repeat revascularization: 9.0% vs. 17.9%; OR 0.36, 95% CI 0.26–0.51; *P* < 0.00001; I^2^ = 70%; MACE: 13.2% vs. 21.5%; OR 0.47, 95% CI 0.35–0.62; P < 0.00001; I^2^ = 68%)(Fig. 5 and 6).

No significant evidence of publication bias was found through the funnel plot.

## Discussion

In this meta-analysis from the comparison between FFR-guided CR and COR in patients with STEMI and multi-vessel disease, we found that FFR-guided CR resulted in low rate of MACE, including all-cause mortality, non-fatal myocardial infarction and repeat revascularization. The reduction of repeat revascularization was similar to that of MACE, which suggested the decreased need for revascularization favored the low incidence of MACE during the follow up period. Moreover, additional FFR-guidance did not significantly increase the rate of all-cause mortality and non-fatal myocardial infarction.

The results of our meta-analysis were in accordance with some previous studies. PRAMI trial [10] showed that angiography guided complete revascularization in patients with STEMI and multi-vessel disease during the primary PCI, using the criteria of percentage diameter stenosis>50% in one view for non-culprit lesion treatment, significantly decreased the rate of MACE and repeat revascularization without the reduction of the rate of all-cause mortality, as compared to COR. With the treatment criteria of non-culprit lesion changed into percentage diameter stenosis>70% in one view or>50% in two views, CVLPRIT trial [21] reported that angiography guided complete revascularization (during primary PCI: 73%; during staged PCI: 27%) was merely associated with low rate of MACE and repeat revascularization, compared with COR. Nevertheless, as the same as PRAMI trial, the risk of all-cause mortality was not changed.

In consideration of the Dissociation Between Angiographic results and Clinical outcomes in coronary artery Disease [22], FFR was gradually considered to be a good measurement for making decision on treating or not treating the coronary artery lesion, based on its high sensitivity and specificity in identification of ischemia [23]. In stead of the FFR threshold value of 0.75 [23], a FFR value of >0.8 suggested a non-ischemic lesion and good clinical outcome [24]. The results from FAME II trial at 3 years follow-up, demonstrated FFR-guided PCI in patients with stable coronary artery disease to lower mortality rate and the rate of re-infarction, compared to angiography-guided PCI [25]. A meta-analysis showed that FFR-guided PCI in patients with stable coronary artery disease was associated with significantly lower rate of re-infarction when compared to angiography-guided PCI [26]. Beside the patients with stable coronary artery disease, FFR measurement could be used effectively and safely in patients with acute myocardial infarction [14]. The data from a meta-analysis further confirmed the effectiveness and safety of FFR measurement in patients with acute coronary syndrome [27].

Ghani trial was the first RCTs of FFR measurement in patients with STEMI and multi-vessel disease. However, the result of this trial did not support FFR-guided CR early after primary PCI (described in Table 1) because of the high rate of the mortality and re-infarction [17]. Subsequently, as a relatively large trial, DANAMI-3-PRIMULTI [19] showed FFR-guided CR with staged PCI strategy (2 days after primary PCI and before discharge) might favor the reduction of MACE not the all-cause mortality. However, the latest large and multi-center trial, COMPARE ACUTE trial [20] revealed FFR-guided CR (during index PCI procedure: 83%; during index hospitalization: 17%) could not only reduce the rate of MACE, also numerically decrease the incidence of death from any cause without statistically difference.

Our meta-analysis was consistent to another two meta-analyses [28, 29], they confirmed that CR (including angiography-guided and FFR-guided) could merely decrease the incident of MACE and repeat revascularization, not the hard end point (all-cause mortality). Intriguingly, the percentage of all-cause mortality in FFR guided complete revascularization group was 1.3% in the Compare Acute trial and 4.7% in the DANAMI-3–PRIMULTI trial, with 50 and 31% of non–infarct-related lesions with a negative FFR value respectively. Probably, these good results could be derived from the accurate choose of revascularization. Additionally, our data also supported the reliability, feasibility and safety of FFR-guided complete revascularization (PCI) during acute phase of STEMI.

### Limitations

There were some limitations in this meta-analysis, including: Firstly, the study population was small, only three RCTs with 1631 patients; Secondly, the study year difference among trials was too much. One was from 2004 to 2007, and others were from 2011 to 2014/2015. Different era might have different technique, different device (like stent type), different concept and different drug, which could affect the result; Thirdly, The cut-off value of FFR was different. The former one study was 0.75 and the latter two studies were 0.80; Finally, all trials included in this meta-analysis were open-label design, which induced potential bias.

## Conclusion

In comparison to COR, among patients with acute STEMI and MVD, FFR-guided functionally CR favored the reduction of the risk of MACE and ischemia-driven repeat revascularization, without a reduction in the rate of all-cause mortality. In the future, further large RCTs are required to investigate whether FFR guidance of complete revascularization significantly affects hard end point (all-cause mortality).

## Additional files

Additional file 1: **Figure S1.** Risk of bias graph. (PDF 789 kb)

Additional file 2: **Figure S2.** Risk of bias summary. (PDF 1453 kb)

## Abbreviations

COR: Culprit-only revascularization
CR: Complete revascularization
FFR: Fractional flow reserve
IRA: infarct-related coronary artery
MACE: major adverse cardiovascular event
MVD: Multi-vessel disease
PCI: percutaneous coronary intervention
STEMI: ST-segment elevation myocardial infarction
