# Supplementary figures and images for: Fractional flow reserve-guided complete revascularization versus culprit-only revascularization in acute ST-segment elevation myocardial infarction and multi-vessel disease patients: a meta-analysis and systematic review
Source: BMC Cardiovasc Disord. 2019 Mar 1;19:49. doi: 10.1186/s12872-019-1022-6 (PMC6397458; doi:10.1186/s12872-019-1022-6)

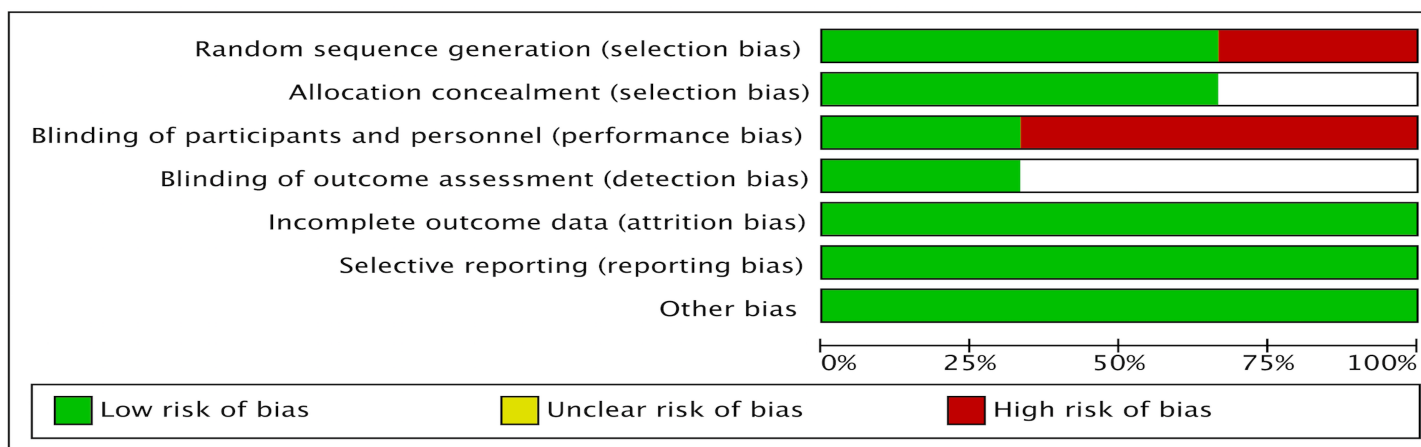

Supplement: Supplementary file 1 — Figure S1. Risk of bias graph. (PDF 789 kb) [file 12872_2019_1022_MOESM1_ESM.pdf]
