# Supplementary material for: Fractional flow reserve-guided complete revascularization versus culprit-only revascularization in acute ST-segment elevation myocardial infarction and multi-vessel disease patients: a meta-analysis and systematic review
Source: BMC Cardiovasc Disord. 2019 Mar 1;19:49. doi: 10.1186/s12872-019-1022-6 (PMC6397458; doi:10.1186/s12872-019-1022-6)
Supplement: Supplementary file 2 — Figure S2. Risk of bias summary. (PDF 1453 kb) [file 12872_2019_1022_MOESM2_ESM.pdf]

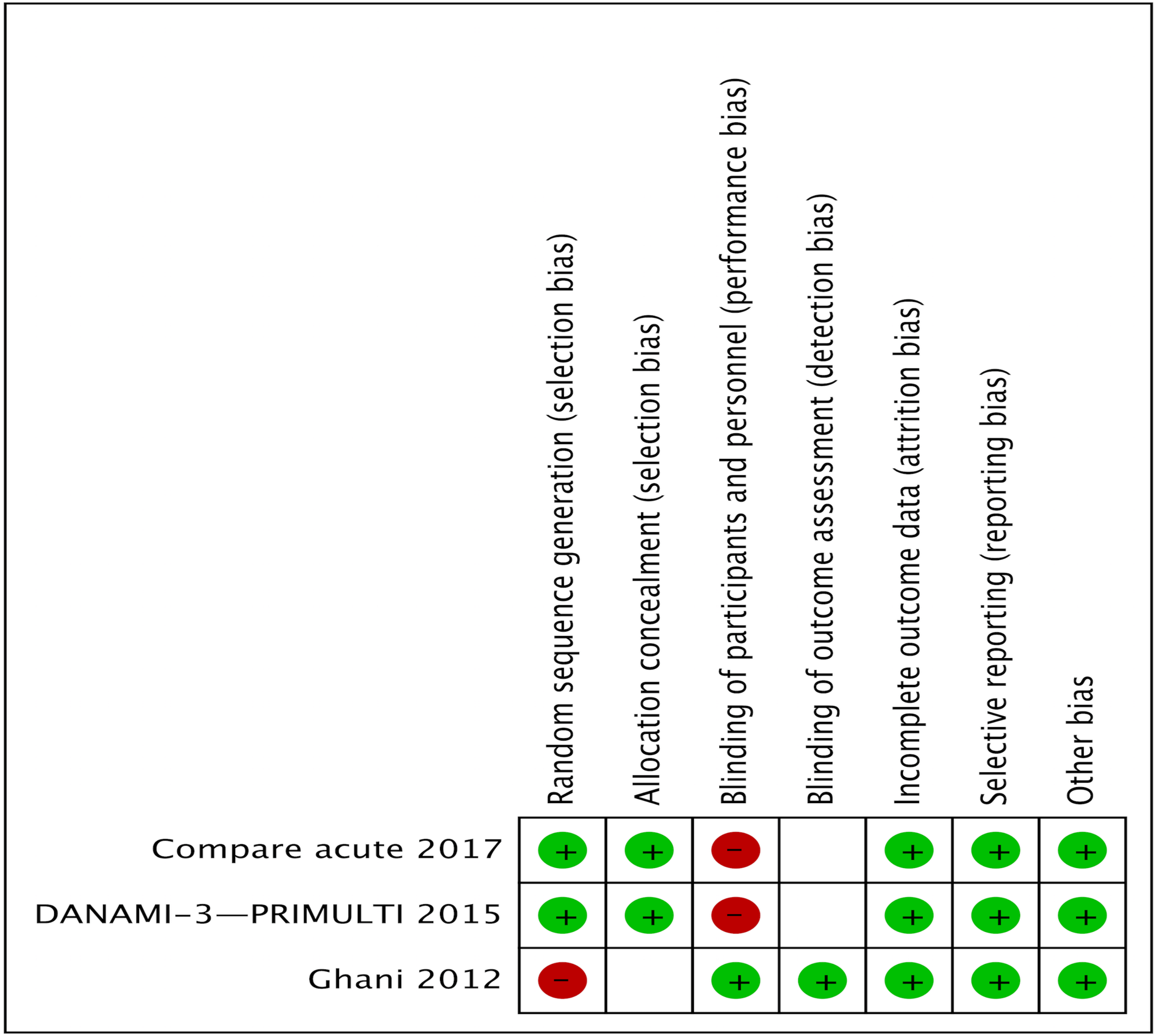
Random sequence generation (selection bias)
Allocation concealment (selection bias)
Blinding of participants and personnel (performance bias)
Blinding of outcome assessment (detection bias)
Incomplete outcome data (attrition bias)
Selective reporting (reporting bias)
Other bias
Compare acute 2017
DANAMI-3—PRIMULTI 2015
Ghani 2012
